# Survival of men with metastatic hormone-sensitive prostate cancer and adrenal-permissive *HSD3B1* inheritance

**DOI:** 10.1172/JCI183583

**Published:** 2024-09-17

**Authors:** Nima Sharifi, Robert Diaz, Hui-Ming Lin, Evan Roberts, Lisa G. Horvath, Andrew Martin, Martin R. Stockler, Sonia Yip, Vinod V. Subhash, Neil Portman, Ian D. Davis, Christopher J. Sweeney

**Affiliations:** 1 Desai Sethi Urology Institute and; 2 Sylvester Comprehensive Cancer Center, University of Miami Miller School of Medicine, Miami, Florida, USA.; 3 Advanced Prostate Cancer Group, Garvan Institute of Medical Research, Darlinghurst, Sydney, New South Wales, Australia.; 4 School of Clinical Medicine, UNSW, Sydney, New South Wales, Australia.; 5 Chris O’Brien Lifehouse, Sydney, New South Wales, Australia.; 6 Sydney Medical School, University of Sydney, Sydney, New South Wales, Australia.; 7 Australian and New Zealand Urogenital and Prostate Cancer Trials Group (ANZUP), Sydney, New South Wales, Australia.; 8 National Health and Medical Research Council Clinical Trials Centre, University of Sydney, Sydney, New South Wales, Australia.; 9 Centre for Clinical Research, University of Queensland, Herston, Queensland, Australia.; 10 Eastern Health Clinical School, Monash University, Melbourne, Victoria, Australia.; 11 Department of Cancer Services, Eastern Health, Melbourne, Victoria, Australia.; 12 South Australian Immunogenomics Cancer Institute, University of Adelaide, Adelaide, South Australia, Australia.

**Keywords:** Endocrinology, Oncology, Prostate cancer, Sex hormones

## Abstract

**BACKGROUND:**

Metastatic hormone-sensitive prostate cancer (mHSPC) is androgen dependent, and its treatment includes androgen deprivation therapy (ADT) with gonadal testosterone suppression. Since 2014, overall survival (OS) has been prolonged with addition of other systemic therapies, such as adrenal androgen synthesis blockers, potent androgen receptor blockers, or docetaxel, to ADT. *HSD3B1* encodes the rate-limiting enzyme for nongonadal androgen synthesis, 3β-hydroxysteroid dehydrogenase-1, and has a common adrenal-permissive missense-encoding variant that confers increased synthesis of potent androgens from nongonadal precursor steroids and poorer prostate cancer outcomes.

**METHODS:**

Our prespecified hypothesis was that poor outcome associated with inheritance of the adrenal-permissive *HSD3B1* allele with ADT alone is reversed in patients with low-volume (LV) mHSPC with up-front ADT plus addition of androgen receptor (AR) antagonists to inhibit the effect of adrenal androgens. *HSD3B1* genotype was obtained in 287 patients with LV disease treated with ADT + AR antagonist only in the phase III Enzalutamide in First Line Androgen Deprivation Therapy for Metastatic Prostate Cancer (ENZAMET) trial and was associated with clinical outcomes.

**RESULTS:**

Patients who inherited the adrenal-permissive *HSD3B1* allele had more favorable 5-year clinical progression-free survival and OS when treated with ADT plus enzalutamide or ADT plus nonsteroidal antiandrogen compared with their counterparts who did not have adrenal-permissive *HSD3B1* inheritance. *HSD3B1* was also associated with OS after accounting for known clinical variables. Patients with both genotypes benefited from early enzalutamide.

**CONCLUSION:**

These data demonstrated an inherited physiologic driver of prostate cancer mortality is associated with clinical outcomes and is potentially pharmacologically reversible.

**FUNDING:**

National Cancer Institute, NIH; Department of Defense; Prostate Cancer Foundation, Australian National Health and Medical Research Council.

## Abstract 2

This work shows that poor outcomes in men treated with ADT who inherit the adrenal-permissive HSD3B1 allele are reversible with upfront use of direct AR blockade.

## Introduction

Prostate cancer has long been recognized to be an androgen-dependent disease, with some cancers being more dependent than others. Androgen deprivation therapy (ADT) with gonadal testosterone suppression has therefore been the standard of care for up-front treatment of metastatic prostate cancer for over 80 years (1). The up-front treatment for advanced disease has become highly complex, with treatment intensification having been integrated as part of the standard of care for metastatic hormone-sensitive prostate cancer (mHSPC), including therapies such as chemotherapy (docetaxel), androgen receptor (AR) antagonists (enzalutamide, apalutamide, and darolutamide), and adrenal androgen synthesis inhibition (abiraterone) (2). The complexity of up-front treatment will continue to increase as other treatments are also introduced in this setting and as systemic therapies are given in earlier disease states that also necessitate longer treatment durations. There is frequently an absence of consensus on which patients should get which combinations. Furthermore, there is currently very limited and no standard role for germline genetic testing in selecting the best up-front therapy for an individual man with prostate cancer, nor is there any role for integrating information on variations in an individual’s physiology (3). Ongoing efforts include those to test the potential role for poly-ADP ribose polymerase inhibition on the basis of alterations in DNA damage repair pathway genes (4).

**Figure 1:**
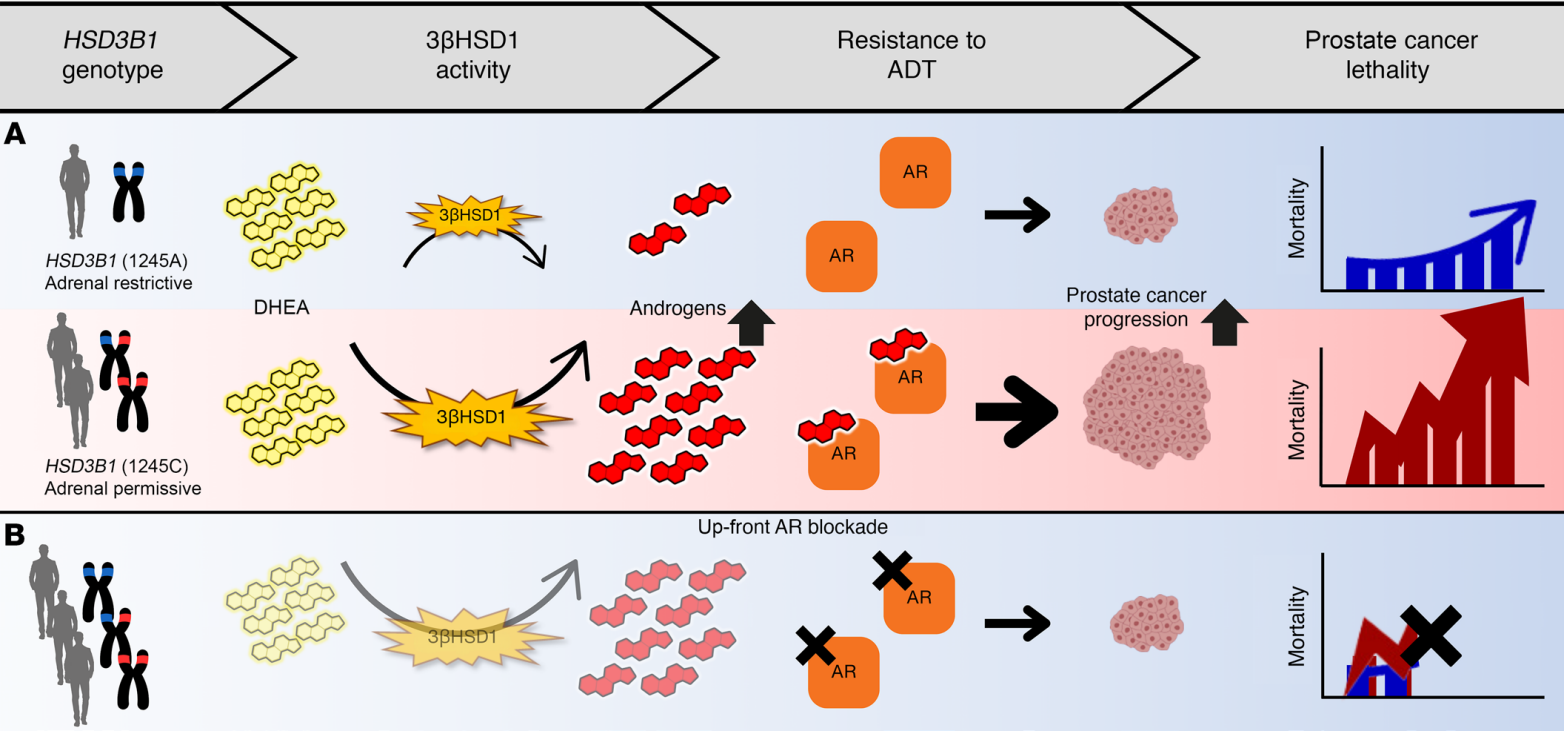
*HSD3B1* genotype regulates nongonadal androgen synthesis and drives prostate cancer progression following ADT. (**A**) The adrenal-permissive *HSD3B1* (1245C) genotype encodes a more active enzyme, resulting in increased synthesis of nongonadal androgens, leading to more rapid progression to castration-resistant prostate cancer and increased prostate cancer mortality. (**B**) Up-front AR blockade given with ADT in the ENZAMET study is expected to prevent worse clinical outcomes and mortality associated with adrenal-permissive allele inheritance.

Inhibition of the gonadal androgen axis with ADT leaves the adrenal androgen axis intact as the major source for extragonadal androgens. The most abundant steroid in circulation is dehydroepiandrosterone (DHEA; in free and sulfated forms), which is made by human adrenals and is converted in prostate cancer tissues to potent androgens, i.e., testosterone and dihydrotestosterone, which in turn drive ADT resistance. The first and rate-limiting enzyme that converts DHEA to potent androgens is 3β-hydroxysteroid dehydrogenase-1 (3βHSD1; encoded by *HSD3B1*). The adrenal-permissive *HSD3B1* (1245C) allele (so termed because it enables tumors to use adrenal precursors) is a common missense-encoding variant, resulting in a stable form of the enzyme that increases metabolic flux from DHEA to dihydrotestosterone, and is widely validated to confer less benefit from gonadal testosterone suppression alone across many cohorts of men with prostate cancer from around the world (Figure 1A) (5–12). About half of all men with prostate cancer inherit at least 1 copy of the adrenal-permissive *HSD3B1* allele, which is associated with more rapid ADT resistance and poorer survival in men with nonmetastatic disease and low-volume (LV) metastatic disease (6, 8). In mHSPC, LV disease is generally more AR dependent, with less benefit from chemotherapy, compared with high-volume (HV) disease and evidence of a higher AR gene expression profile (13–15). Furthermore, in a study of over 5,200 men that also includes those with localized disease, adrenal-permissive *HSD3B1* allele homozygosity is associated with a higher risk of prostate cancer death, thus making it the most common monogenic link to prostate cancer mortality (16, 17).

We hypothesized that up-front pharmacologic blockade by use of AR antagonists alone with ADT would reverse the poor outcomes and survival associated with the adrenal-permissive *HSD3B1* allele in patients with LV metastatic disease (Figure 1B). Enzalutamide in First Line Androgen Deprivation Therapy for Metastatic Prostate Cancer (ENZAMET) is a practice-changing randomized trial that used treatment with direct AR blockade plus ADT in both arms (18, 19). The treatment comparison was use of a more effective AR blocker (enzalutamide) versus a weaker nonsteroidal antiandrogen (NSAA). The hypothesis was prespecified in a grant funded in 2019 (Congressionally Directed Medical Research Programs Department of Defense W81XWH-20-1-0137) and included *HSD3B1* genotype categorization and determination of whether poor outcomes for adrenal-permissive *HSD3B1* inheritance could be reversed with up-front blockade of nongonadal androgens.

## Results

**Figure 2:**
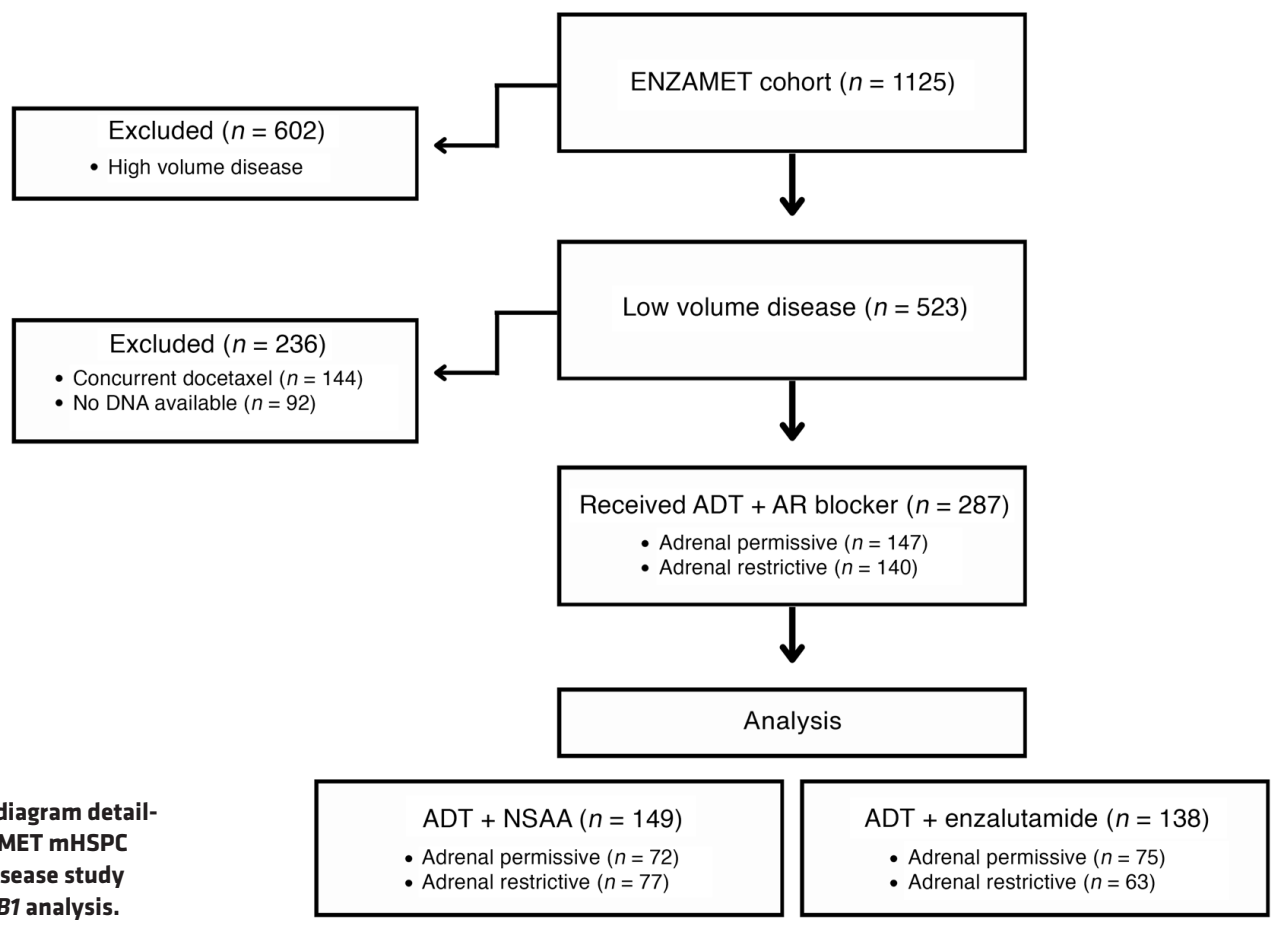
CONSORT diagram detailing the entire ENZAMET mHSPC cohort and the LV disease study cohort for the *HSD3B1* analysis.

**Figure 3:**
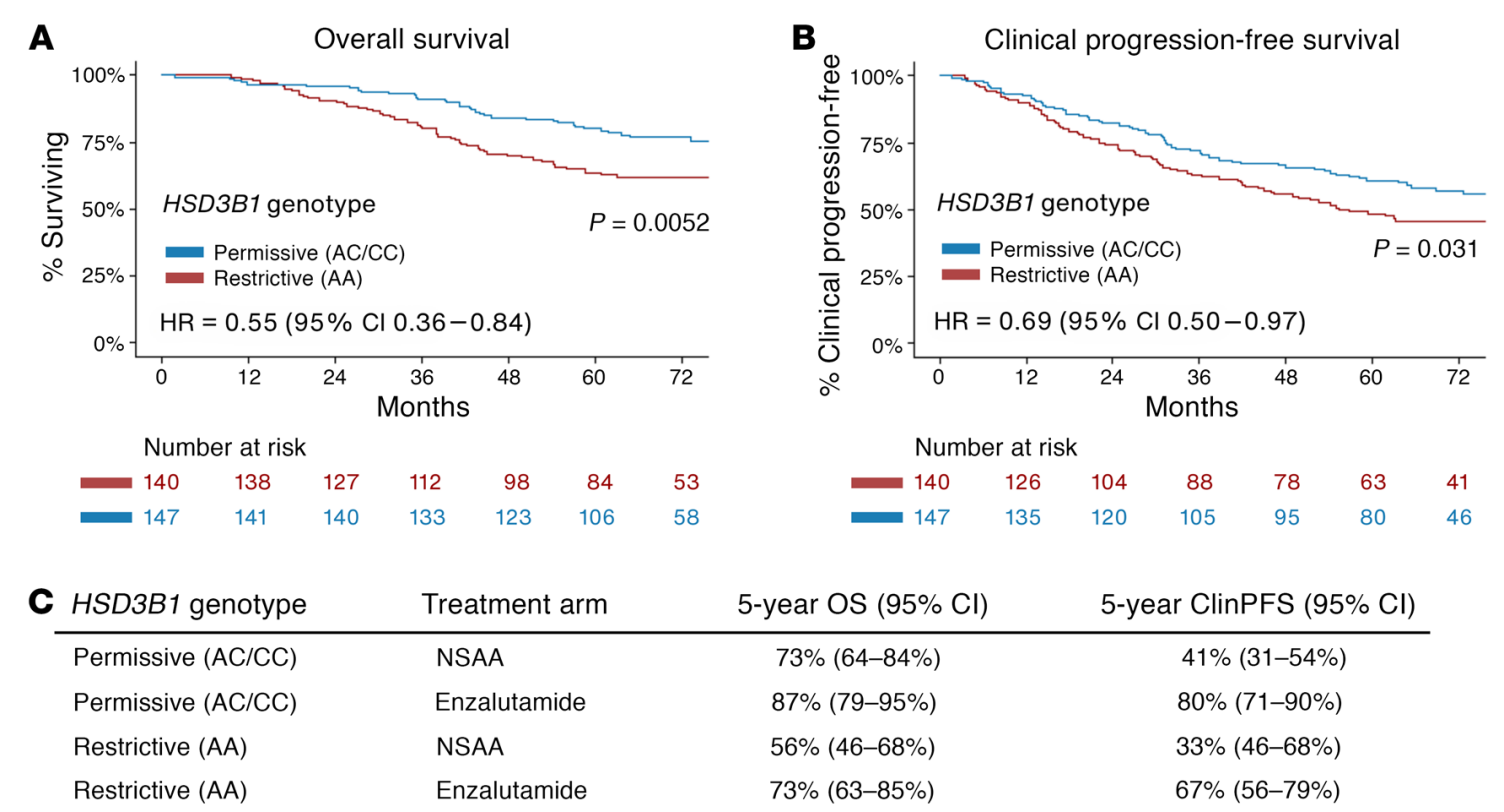
Up-front AR blockade reverses the poor outcomes associated with adrenal-permissive allele inheritance. (**A** and **B**) Kaplan-Meier plots showing OS (**A**) and cPFS (**B**) according to *HSD3B1* genotype. (**C**) Five-year OS and cPFS by treatment arm and *HSD3B1* genotype.

Baseline characteristics of the study cohort are in Tables 1 and 2, and the CONSORT diagram is in Figure 2. Of the 287 patients with LV metastatic disease on the trial who were treated with ADT + AR blocker on either arm, had DNA available, and did not receive concurrent docetaxel, 147 inherited the adrenal-permissive genotype, and 140 inherited the adrenal-restrictive genotype. At progression to castration-resistant prostate cancer (CRPC), patients were treated with standard-of-care therapies. Of the entire cohort treated with ADT + NSAA, 85% received life-prolonging therapy (enzalutamide, abiraterone, and chemotherapy), and other treatments could have included antiandrogen withdrawal therapy (19). Thus, a large majority of patients in this study were treated with enzalutamide either for mHSPC or for metastatic CRPC. Although prior data showed men treated with ADT alone and who had adrenal-permissive *HSD3B1* inheritance had poorer outcomes (6, 8), we observed in the ENZAMET trial that patients with adrenal-permissive *HSD3B1* inheritance (Figure 1A) (6, 8) treated with ADT + enzalutamide or less potent antiandrogens (mostly bicalutamide) had better overall survival (OS) compared with patients with adrenal-restrictive *HSD3B1* inheritance (HR = 0.55; 95% CI = 0.36–0.84; *P* = 0.0052) (Figure 3A). Similarly, clinical progression-free survival (cPFS) also favored the adrenal-permissive group (HR = 0.69; 95% CI = 0.50–0.97; *P* = 0.031) (Figure 3B). The lower HR (OS) than HR (cPFS) may relate to the high rate of enzalutamide therapy at the time of progression to castration resistance in the ADT + NSAA arm (19).

In the enzalutamide + ADT arm, *HSD3B1* comparisons for cPFS showed HR = 0.54 (95% CI = 0.29–1.00; *P* = 0.046) and for OS showed HR = 0.51 (95% CI = 0.25–1.01; *P* = 0.066). In the ADT + NSAA arm, comparisons for cPFS showed HR = 0.85 (95% CI = 0.57–1.26; *P* = 0.42) and for OS showed HR = 0.60 (95% CI = 0.36–1.02; *P* = 0.056) (Supplemental Figures 1 and 2; supplemental material available online with this article; https://doi.org/10.1172/JCI183583DS1). The 5-year cPFS and OS for both genotypes and treatment arms are shown in Figure 3C. Multivariable Cox regression analysis showed that adrenal-permissive *HSD3B1* was associated with better OS and cPFS when treated with ADT plus an antiandrogen even after accounting for other clinical variables (Tables 3 and 4). Exploratory OS and cPFS outcomes with 0, 1, and 2 adrenal-permissive *HSD3B1* alleles are shown in Supplemental Figures 3 and 4 and limited because of smaller numbers of patients for the homozygous adrenal-permissive groups.

## Discussion

The missense-encoding adrenal-permissive *HSD3B1* allele, which is present in about half of all men, enables prostate cancer intratumoral androgen biosynthesis from nongonadal precursor steroids, thus linking this genetically driven mechanism to poorer clinical outcomes across multiple cohorts and settings, including localized and metastatic disease. A major question arising from the clinical data to date is whether these poor clinical outcomes with adrenal-permissive *HSD3B1* allele inheritance are pharmacologically reversible by blocking the effects of augmented intratumoral androgen biosynthesis or if the poor outcomes persist despite hormonal therapy intensification. The data presented in this paper indicate that up-front blockade of nongonadal androgens with direct AR antagonism not only appears to reverse the poor outcomes driven by adrenal-permissive allele inheritance reported in other cohorts but also may improve outcomes beyond those for men who do not have adrenal-permissive allele inheritance (i.e., adrenal-restrictive inheritance). This could be biologically plausible if prostate cancers in men with the adrenal-permissive *HSD3B1* genotype are more (nongonadal) androgen dependent compared with adrenal-restrictive *HSD3B1* tumors, with the latter having a decreased capacity to use and depend on extragonadal androgens. Tumors that harbor the adrenal-permissive *HSD3B1* genotype and have an apparent nongonadal androgen dependency could use either adrenal androgens or de novo androgen biosynthesis in the tumors from cholesterol. Both adrenal and de novo pathways require the same enzymatic steps catalyzed by 3βHSD1 and are necessary to make testosterone or dihydrotestosterone (20). The interaction between *HSD3B1* genotype and clinical outcomes in this study is apparent both with weaker (NSAA) and stronger (enzalutamide) AR antagonists. Further, an augmented effect on reversal of poor adrenal-permissive inheritance–associated outcomes on cPFS appears to be conferred to a greater extent by enzalutamide than by the weaker antiandrogen. It should also be highlighted that the improved outcomes with early enzalutamide were still observed in patients with the adrenal-restrictive genotype.

ENZAMET is the third randomized phase III trial in mHSPC analyzed for outcomes by *HSD3B1* genotype. Analysis of E3805, a trial of ADT plus or minus docetaxel and no mandate of long-term antiandrogen with ADT, demonstrated worse outcomes with ADT for adrenal-permissive *HSD3B1* inheritance in LV disease but no significant difference by *HSD3B1* genotype in HV disease (8). ARCHES is a trial of ADT plus or minus enzalutamide (21). However, of those who underwent *HSD3B1* genotyping with a median follow-up of about 46 months, less than 10% of patients with LV disease in the ADT + enzalutamide arm experienced progression (22). Thus, the *HSD3B1* analysis in ARCHES is limited by a small number of events for LV disease because of shorter follow-up. The ARCHES results are driven by clinical events in HV disease, in which there were no significant cPFS differences by *HSD3B1* genotype (22). Nevertheless, the common finding between E3805 and ARCHES is that there is no significant association between *HSD3B1* genotype and clinical progression in HV mHSPC.

It is possible that these data are also influenced by somatic mutations that are known to occur in mHSPC, including in PTEN, Rb1, and p53 (23, 24). However, studies to date have not identified any profound associations between somatic genetic alterations and germline *HSD3B1* in prostate cancer, and thus, a bias because of co-occurring somatic mutations is unlikely (25, 26). Instead, adrenal-permissive *HSD3B1* genotypes are associated with increased cell cycle regulation and AR signaling signatures (25, 26). However, other tumor-level alterations in *HSD3B1* are known to occur, including somatic mutations (5), phosphorylation of the 3βHSD1 protein (27), regulation by cancer-associated fibroblasts (28), and regulation by hypoxia-dependent mechanisms (29, 30), which may contribute to clinical outcomes.

It is unknown whether patients with LV mHSPC and the adrenal-permissive allele treated with abiraterone, an inhibitor of gonadal and extragonadal androgen synthesis inhibition, would experience the same effect observed with antiandrogens in this study. Notably, abiraterone has a steroidal structure and is susceptible to metabolism by enzymes that usually metabolize endogenous steroids. Principally, the steroid A and B rings of abiraterone are identical to DHEA, which makes both substrates of 3βHSD1. Abiraterone is converted by 3βHSD1 to Δ^4^-abiraterone (31) and 5α-abiraterone (32), which have AR antagonist and partial AR agonist activity, respectively. Nevertheless, the ultimate clinical consequences of lower or higher abiraterone metabolism with the adrenal-restrictive or adrenal-permissive *HSD3B1* alleles are not known (33).

Limitations of this analysis include the exclusion of the patients chosen for docetaxel. The hypothesis pertained to patients treated with ADT + NSAA or enzalutamide without docetaxel. Men for whom docetaxel was selected (physician’s choice) in general had worse prognostic disease (19). The absence of an ADT-alone cohort in this trial precludes the ability to directly observe whether the reversal of survival outcomes by *HSD3B1* genotype is real or due to bias associated with the genotypes in the data set. However, an accounting for other known clinical variables does not appear to suggest a bias (Tables 2 and 4). Additional analyses in other studies with ADT-alone comparator arms should resolve this question.

Genetic biomarkers have not been incorporated into practice-changing clinical trials for up-front treatment of metastatic prostate cancer to date. Rational integration of genetic biomarkers is necessary for patient selection as additional combination therapy trials are developed and treatments move into earlier disease settings, which also necessitate longer treatment durations that must be balanced by a consideration of the resultant increase in adverse effects. The prostate cancer mortality data associated with *HSD3B1* (16, 17), combined with the pharmacologic actionability inferred by this study, together suggest that *HSD3B1* should also be interrogated in ongoing hormonal therapy studies in earlier disease states, including PROTEUS (NCT03767255), ENZARAD (NCT02446444), DASL-HiCaP (NCT0436353), NRG-GU008 (NCT04134260), NRG-GU009 (NCT04513717), and others. As a germline biomarker, *HSD3B1* circumvents the caveats of detecting somatic alterations, which include tumor DNA fraction, tumor heterogeneity, or the potential necessity of invasive tumor biopsies (24, 34, 35). The relative simplicity of obtaining germline DNA combined with an established mechanism through which *HSD3B1* inheritance confers resistance to castration using an extragonadal androgen-dependent mechanism strongly supports integration of *HSD3B1* into prostate cancer clinical trials in development, and thus the potential role of *HSD3B1* in routine clinical care remains to be determined.

Finally, these data further highlight the potential for development of pharmacologic inhibitors against 3βHSD1, particularly for patients who harbor the adrenal-permissive *HSD3B1* allele. The apparent sensitivity of adrenal-permissive *HSD3B1* tumors to up-front, intensified hormonal therapy raises the possibility of even better clinical outcomes with direct 3βHSD1 enzyme inhibitors. Strategies for developmental therapeutics include competitive enzymatic inhibition of 3βHSD1 or inhibition of 3βHSD1 phosphorylation using blockade of the BMX tyrosine kinase (27, 36).

In conclusion, ENZAMET is the first randomized study with sufficient power and follow-up to test the effect of up-front AR blockade in LV mHSPC. Use of NSAA in the control arm enabled testing for the *HSD3B1* genotype interaction with both potent (enzalutamide) and modest (NSAA) blockade of the effects of sustained extragonadal androgen biosynthesis that occurs with the adrenal-permissive *HSD3B1* allele. The data presented in this paper demonstrated that an inherited physiologic driver of prostate cancer mortality, adrenal-permissive *HSD3B1* genotype, which has been shown to be associated with poorer clinical outcomes in nonmetastatic HSPC and mHSPC, is potentially pharmacologically reversible with potent AR antagonism. Together, these data indicate that *HSD3B1* inheritance may drive a fundamental and mechanistic aspect of prostate cancer physiology that is pharmacologically actionable and reversible.

## Methods

### Sex as a biological variable.

Prostate cancer generally affects men. Therefore, this study included only men with prostate cancer.

### Genotyping and definitions.

*HSD3B1* genotyping was performed in 2023 using a method previously described (6, 37). The association between clinical outcomes and the adrenal-permissive genotype (1+ adrenal-permissive alleles) versus adrenal-restrictive genotypes (0 adrenal-permissive alleles) was determined. The cutoff date of the clinical data was the same as for the planned primary OS analysis of the trial (January 19, 2022) (19). OS was defined as time from randomization of the participant until death from any cause or the date of last known follow-up. cPFS was defined as the earliest sign of radiographic progression using the Prostate Cancer Working Group 2 criteria (38) for bone lesions and Response Evaluation Criteria in Solid Tumors (version 1.1) (39) for soft-tissue lesions, symptoms attributable to cancer progression, or initiation of another anticancer treatment for prostate cancer.

### Statistics.

Survival analyses were performed using the Kaplan-Meier method. HRs were estimated by Cox regression. The 5-year time point was used to compare long-term outcomes as median OS was not met for the groups of interest. All statistical analyses were performed using R (version 4.2.3) with the packages survival (version 3.5-7) and survminer (version 0.4.9). *P* < 0.05 was considered statistically significant.

### Study approval.

Written informed consent was obtained from all participants as previously published in the ENZAMET trial. Ethical approval was obtained to test this hypothesis by genotyping germline DNA collected from patients enrolled on ENZAMET, as approved separately in each region.

### Data availability.

The Supporting Data Values are provided in an XLS file.

## Author contributions

NS conceptualized the genetic analysis; HML, LGH, IDD, and CS designed the research study; RD performed genotyping analysis; LGH, AM, MS, SY, VS, NP, IDD, and CS performed research related to the clinical trial; NS wrote the first manuscript draft; and ER and all other authors critically reviewed and contributed to the manuscript.

## Supplementary Material

Supplemental data

ICMJE disclosure forms

Supporting data values

## Figures and Tables

**Table 2:**
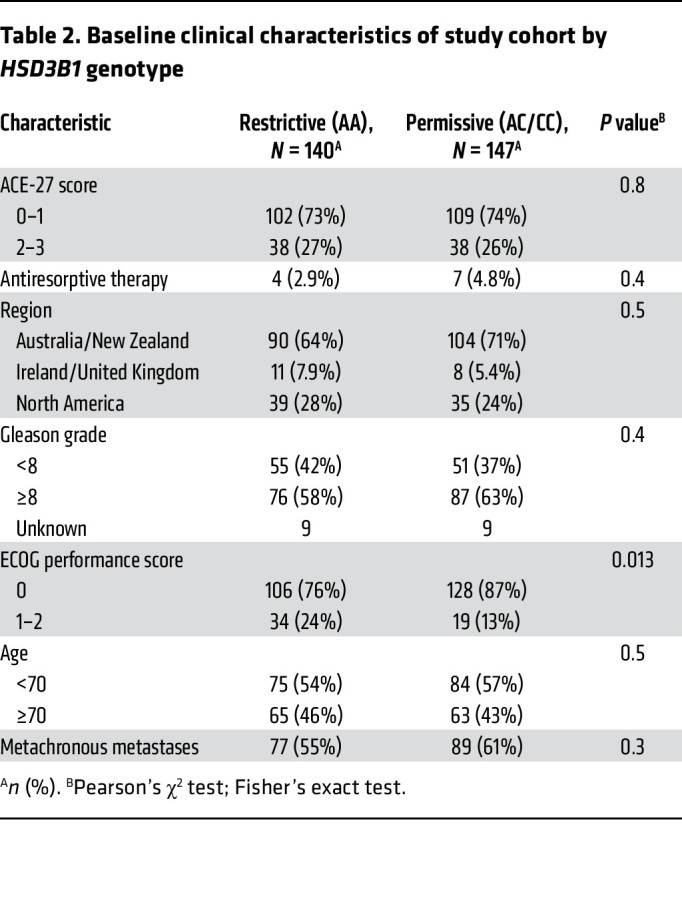
Baseline clinical characteristics of study cohort by *HSD3B1* genotype

**Table 1:**
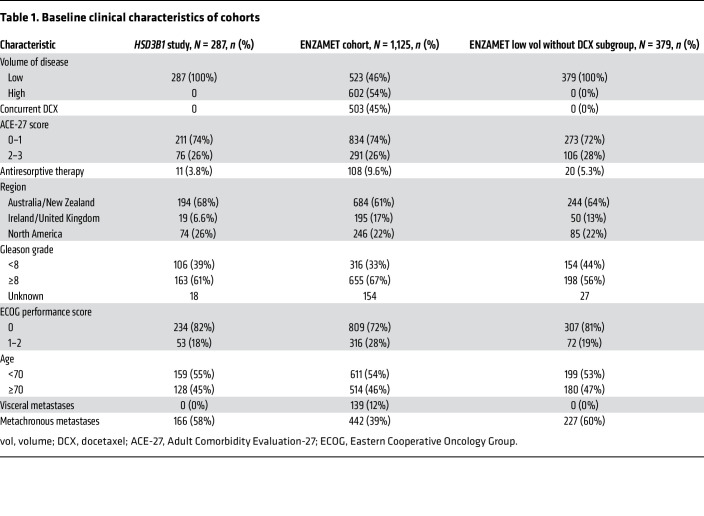
Baseline clinical characteristics of cohorts

**Table 3:**
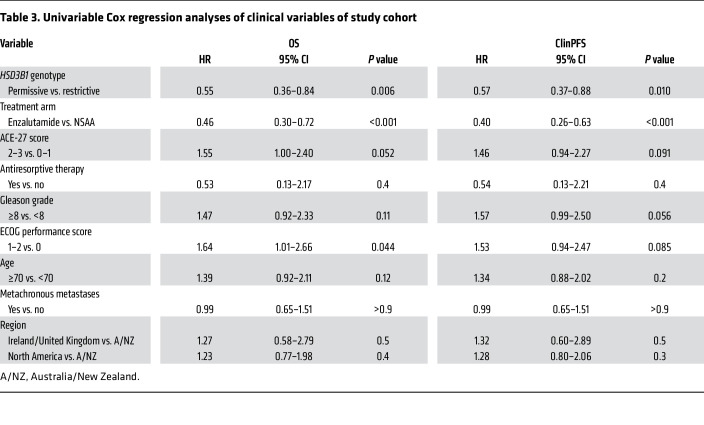
Univariable Cox regression analyses of clinical variables of study cohort

**Table 4:**
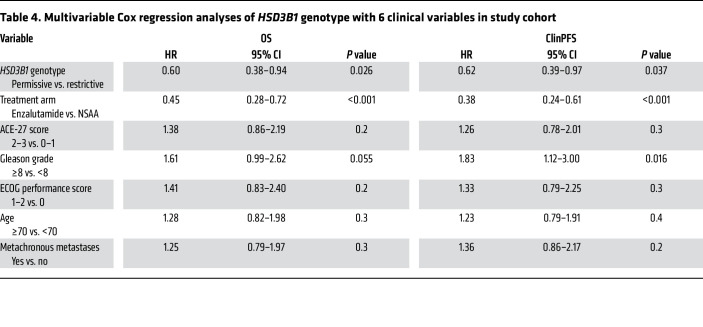
Multivariable Cox regression analyses of *HSD3B1* genotype with 6 clinical variables in study cohort

## Version 1 09/17/2024

Electronic publication

## References

[1] Dai C (2023). Targeting the androgen signaling axis in prostate cancer. J Clin Oncol.

[2] Gillessen S (2023). Management of patients with advanced prostate cancer-metastatic and/or castration-resistant prostate cancer: report of the Advanced Prostate Cancer Consensus Conference (APCCC) 2022. Eur J Cancer.

[3] Hofmann MR (2021). Prostate Cancer Foundation hormone-sensitive prostate cancer biomarker working group meeting summary. Urology.

[4] Martinez Chanza N (2022). Prevalence and clinical impact of tumor BRCA1 and BRCA2 mutations in patients presenting with localized or metastatic hormone-sensitive prostate cancer. Prostate Cancer Prostatic Dis.

[5] Chang KH (2013). A gain-of-function mutation in DHT synthesis in castration-resistant prostate cancer. Cell.

[6] Hearn JWD (2016). *HSD3B1* and resistance to androgen-deprivation therapy in prostate cancer: a retrospective, multicohort study. Lancet Oncol.

[7] Agarwal N (2017). Independent validation of effect of *HSD3B1* genotype on response to androgen-deprivation therapy in prostate cancer. JAMA Oncol.

[8] Hearn JWD (2020). *HSD3B1* genotype and clinical outcomes in metastatic castration-sensitive prostate cancer. JAMA Oncol.

[9] Shiota M (2019). Association of missense polymorphism in *HSD3B1* with outcomes among men with prostate cancer treated with androgen-deprivation therapy or abiraterone. JAMA Netw Open.

[10] Khalaf DJ (2020). *HSD3B1* (1245A>C) germline variant and clinical outcomes in metastatic castration-resistant prostate cancer patients treated with abiraterone and enzalutamide: results from two prospective studies. Ann Oncol.

[11] Lu C (2020). Treatment with abiraterone and enzalutamide does not overcome poor outcome from metastatic castration-resistant prostate cancer in men with the germline homozygous *HSD3B1* c.1245C genotype. Ann Oncol.

[12] Thomas L, Sharifi N (2020). Germline *HSD3B1* genetics and prostate cancer outcomes. Urology.

[13] Kyriakopoulos CE (2018). Chemohormonal therapy in metastatic hormone-sensitive prostate cancer: long-term survival analysis of the randomized phase III E3805 CHAARTED trial. J Clin Oncol.

[14] Hamid AA (2021). Transcriptional profiling of primary prostate tumor in metastatic hormone-sensitive prostate cancer and association with clinical outcomes: correlative analysis of the E3805 CHAARTED trial. Ann Oncol.

[15] Vale CL (2023). Which patients with metastatic hormone-sensitive prostate cancer benefit from docetaxel: a systematic review and meta-analysis of individual participant data from randomised trials. Lancet Oncol.

[16] McKay RR, Nelson T (2024). Adrenal-permissive germline *HSD3B1* allele and prostate cancer outcomes. JAMA Netw Open.

[17] Schiffer L, Sharifi N (2024). Adrenal-permissive *HSD3B1* genotype-an invisible stimulator of prostate cancer mortality. JAMA Netw Open.

[18] Davis ID (2019). Enzalutamide with standard first-line therapy in metastatic prostate cancer. N Engl J Med.

[19] Sweeney CJ (2023). Testosterone suppression plus enzalutamide versus testosterone suppression plus standard antiandrogen therapy for metastatic hormone-sensitive prostate cancer (ENZAMET): an international, open-label, randomised, phase 3 trial. Lancet Oncol.

[20] Hettel D, Sharifi N (2018). *HSD3B1* status as a biomarker of androgen deprivation resistance and implications for prostate cancer. Nat Rev Urol.

[21] Armstrong AJ (2019). ARCHES: a randomized, phase III study of androgen deprivation therapy with enzalutamide or placebo in men with metastatic hormone-sensitive prostate cancer. J Clin Oncol.

[22] Sharifi N (2024). *HSD3B1* genotype and outcomes in metastatic hormone-sensitive prostate cancer with androgen deprivation therapy and enzalutamide: ARCHES. Cell Rep Med.

[23] Hamid AA (2019). Compound genomic alterations of TP53, PTEN, and RB1 tumor suppressors in localized and metastatic prostate cancer. Eur Urol.

[24] Warner EW (2024). Multiregion sampling of de novo metastatic prostate cancer reveals complex polyclonality and augments clinical genotyping. Nat Cancer.

[25] Chen WS (2020). Germline polymorphisms associated with impaired survival outcomes and somatic tumor alterations in advanced prostate cancer. Prostate Cancer Prostatic Dis.

[26] Antonarakis ES (2024). The influence of the germline *HSD3B1* adrenal-permissive variant (c.1100 C) on somatic alteration landscape, transcriptome, and immune-cell infiltration in prostate cancer. J Clin Oncol.

[27] Li X (2023). BMX controls 3βHSD1 and sex steroid biosynthesis in cancer. J Clin Invest.

[28] Cui D (2023). Cancer-associated fibroblast-secreted glucosamine alters the androgen biosynthesis program in prostate cancer via *HSD3B1* upregulation. J Clin Invest.

[29] Qin L (2024). Chronic hypoxia stabilizes 3βHSD1 via autophagy suppression. Cell Rep.

[30] Qin L (2022). Hypoxia-reoxygenation couples 3βHSD1 enzyme and cofactor upregulation to facilitate androgen biosynthesis and hormone therapy resistance in prostate cancer. Cancer Res.

[31] Li Z (2015). Conversion of abiraterone to D4A drives anti-tumour activity in prostate cancer. Nature.

[32] Li Z (2016). Redirecting abiraterone metabolism to biochemically fine tune prostate cancer anti-androgen therapy. Nature.

[33] Alyamani M (2018). *HSD3B1*(1245a>c) variant regulates dueling abiraterone metabolite effects in prostate cancer. J Clin Invest.

[34] Romanel A (2015). Plasma AR and abiraterone-resistant prostate cancer. Sci Transl Med.

[35] Beltran H (2016). Divergent clonal evolution of castration-resistant neuroendocrine prostate cancer. Nat Med.

[36] Qiu Y (2023). A phosphorylation switch controls androgen biosynthesis in prostate cancer. J Clin Invest.

[37] Alyamani M (2023). Approaches to assessing 3β-hydroxysteroid dehydrogenase-1. Methods Enzymol.

[38] Scher HI (2008). Design and end points of clinical trials for patients with progressive prostate cancer and castrate levels of testosterone: recommendations of the Prostate Cancer Clinical Trials Working Group. J Clin Oncol.

[39] Eisenhauer EA (2009). New response evaluation criteria in solid tumours: revised RECIST guideline (version 1.1). Eur J Cancer.

