# Supplementary figures and images for: Survival of men with metastatic hormone-sensitive prostate cancer and adrenal-permissive HSD3B1 inheritance
Source: J Clin Invest. 2024 Sep 17;134(18):e183583. doi: 10.1172/JCI183583 (PMC11405037; doi:10.1172/JCI183583)

Supplemental Figures

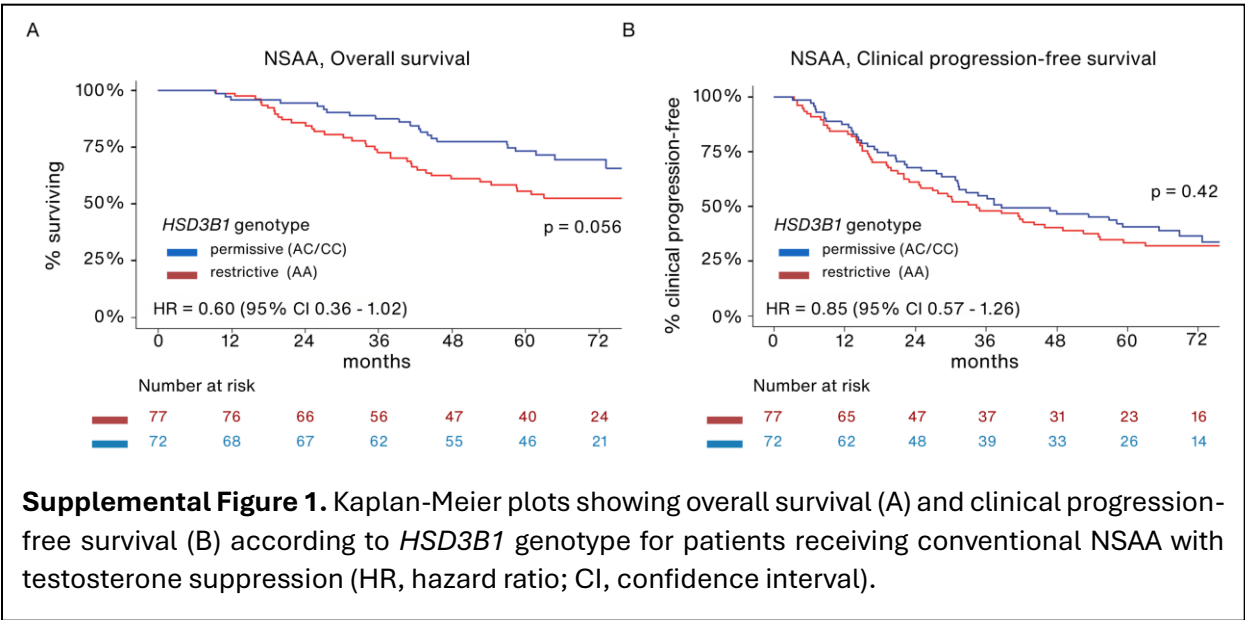

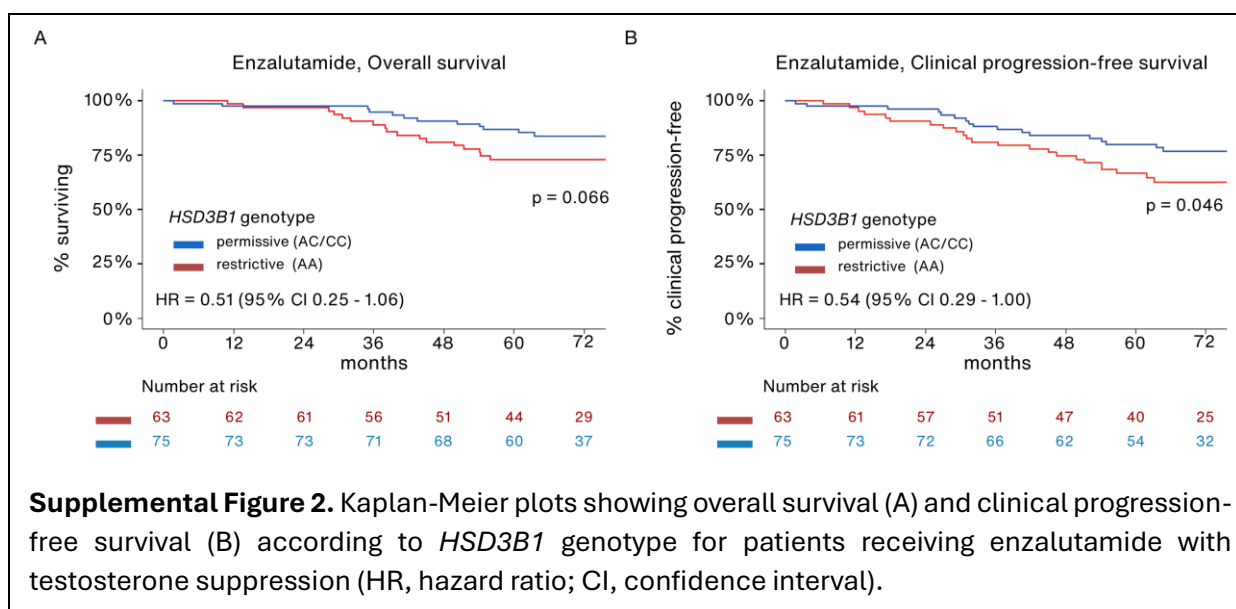

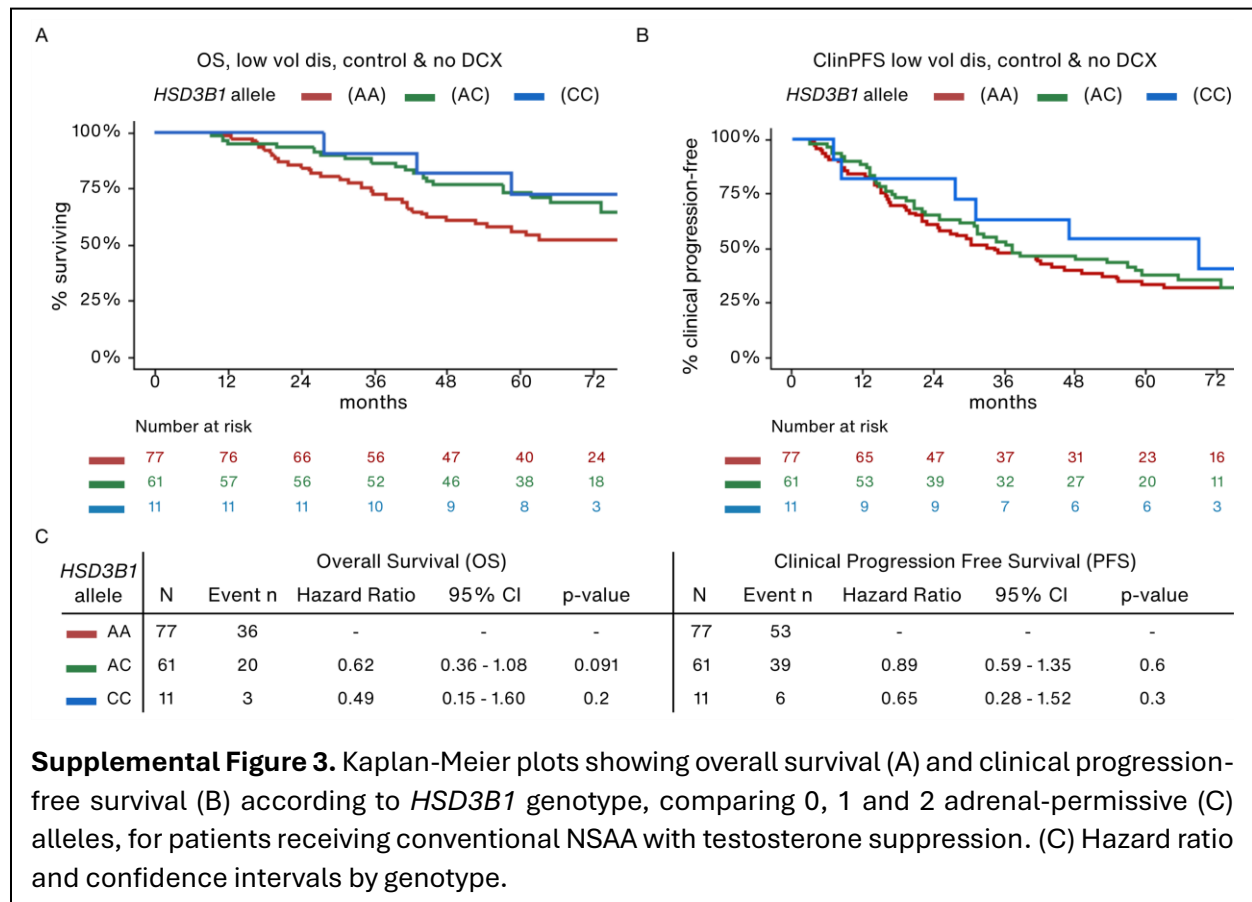

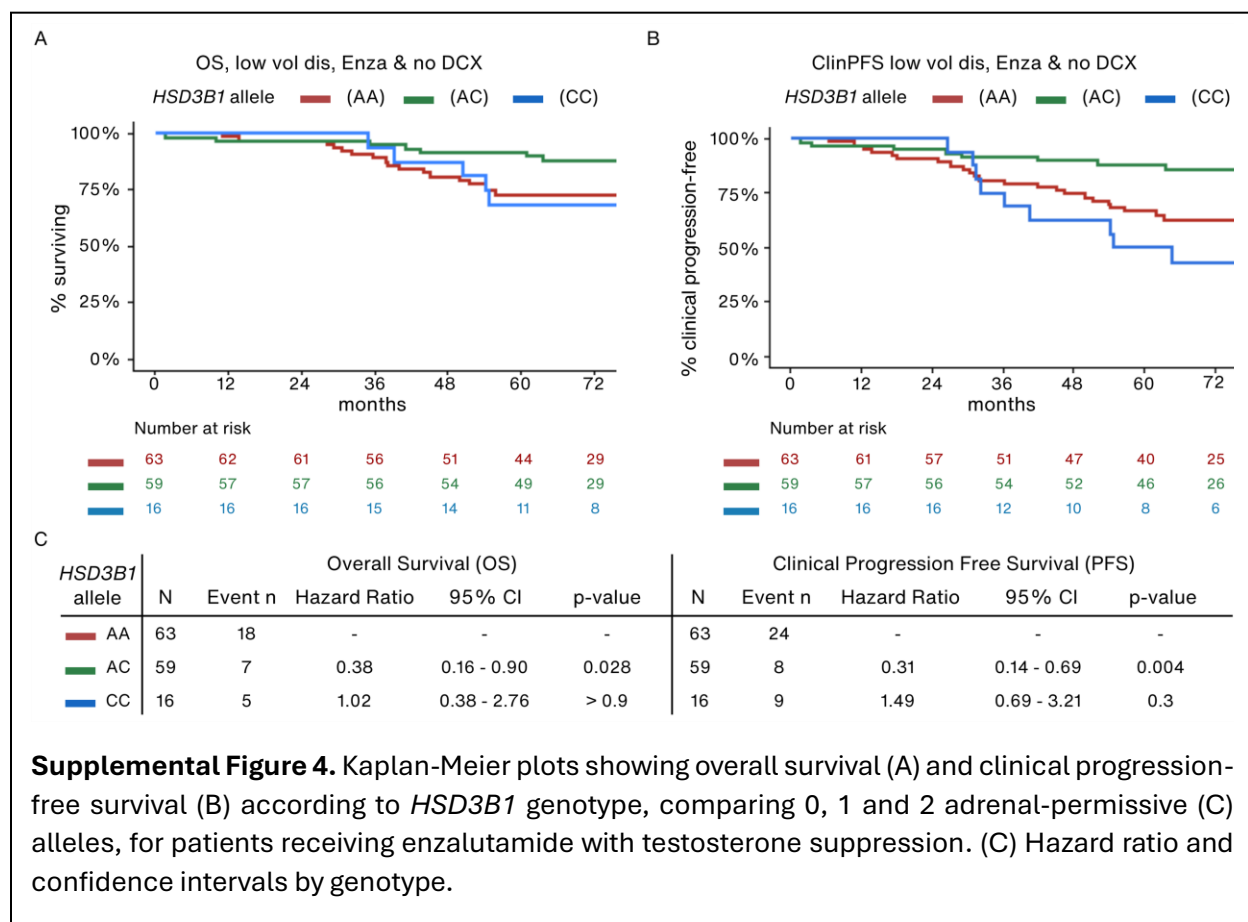

Supplement: Supplemental data [file jci-134-183583-s144.pdf]
